# Falls and consequent injuries in hospitalized patients: effects of an interdisciplinary falls prevention program

**DOI:** 10.1186/1472-6963-6-69

**Published:** 2006-06-07

**Authors:** René Schwendimann, Hugo Bühler, Sabina De Geest, Koen Milisen

**Affiliations:** 1 Institute of Nursing Science, University of Basel, Bernoullistrasse 28, 4056 Basel, Switzerland; 2 Stadtspital Waid Zürich, Zürich, Switzerland; 3 Center for Health Services and Nursing Research, Katholieke Universiteit Leuven, Leuven, Belgium; 4 Department of Geriatrics, University Hospitals of Leuven, Leuven, Belgium

## Abstract

**Background:**

Patient falls in hospitals are common and may lead to negative outcomes such as injuries, prolonged hospitalization and legal liability. Consequently, various hospital falls prevention programs have been implemented in the last decades. However, most of the programs had no sustained effects on falls reduction over extended periods of time.

**Methods:**

This study used a serial survey design to examine in-patient fall rates and consequent injuries before and after the implementation of an interdisciplinary falls prevention program (IFP) in a 300-bed urban public hospital. The population under study included adult patients, hospitalized in the departments of internal medicine, geriatrics, and surgery. Administrative patient data and fall incident report data from 1999 to 2003 were examined and summarized using frequencies, proportions, means and standard deviations and were analyzed accordingly.

**Results:**

A total of 34,972 hospitalized patients (mean age: 67.3, SD ± 19.3 years; female 53.6%, mean length of stay: 11.9 ± 13.2 days, mean nursing care time per day: 3.5 ± 1.4 hours) were observed during the study period. Overall, a total of 3,842 falls affected 2,512 (7.2%) of the hospitalized patients. From these falls, 2,552 (66.4%) were without injuries, while 1,142 (29.7%) falls resulted in minor injuries, and 148 (3.9%) falls resulted in major injuries. The overall fall rate in the hospitals' patient population was 8.9 falls per 1,000 patient days. The fall rates fluctuated slightly from 9.1 falls in 1999 to 8.6 falls in 2003. After the implementation of the IFP, in 2001 a slight decrease to 7.8 falls per 1,000 patient days was observed (p = 0.086). The annual proportion of minor and major injuries did not decrease after the implementation of the IFP. From 1999 to 2003, patient characteristics changed in terms of slight increases (female gender, age, consumed nursing care time) or decreases (length of hospital stay), as well as the prevalence of fall risk factors increased up to 46.8% in those patients who fell.

**Conclusion:**

Following the implementation of an interdisciplinary falls prevention program, neither the frequencies of falls nor consequent injuries decreased substantially. Future studies need to incorporate strategies to maximize and evaluate ongoing adherence to interventions in hospital falls prevention programs.

## Background

Patient falls in hospitals are common and affect approximately 2% to 17% of patients during their hospital stay [1-5]. Fall rates vary from 1.4 up to 17.9 falls per 1,000 patient days depending on hospital type and patient populations [5-17]. Fall related injuries occur in 15% to 50% of the patients, including major injuries such as fractures or lacerations in 1% to 10% [1,6,8,9,13-15,18-21]. Furthermore, falls may lead to fear of falling with subsequent activity restriction [22,23], prolonged hospital stay [24], and legal liability [25]. Various hospital falls prevention programs have been implemented in the last decades [26,27]. Unfortunately, none of these studies has demonstrated a sustained effect over years [26]. In one study, a 25% reduction of falls-related injuries was reported over a 5 year period following the implementation of a prevention program [28]. In 1999, a nurse led falls prevention program implemented in our hospital showed decreasing multiple falls [29]. Consequently, the hospital management launched the development and implementation of an interdisciplinary falls prevention program in 2000 in the departments of internal medicine, geriatrics and surgery. The present study aimed to examine in-patient fall rates and consequent injuries before and after the implementation of the interdisciplinary falls prevention program.

## Methods

### Design, setting and sample

This observational study used a serial survey design and was conducted from January 1^st ^in 1999 to December 31^st ^in 2003 in an urban public teaching hospital in the City of Zurich in Switzerland. The 300-bed hospital provides medical services for 160,000 inhabitants and includes three clinical departments: 1) internal medicine (122 beds), 2) surgery (100 beds), and 3) geriatrics (78 beds). The population observed consisted of adult patients (18 years and older), hospitalized for more than 24 hours in one of the three departments. Patients of the emergency department and intensive care unit were not included. The study was approved by the ethical review board of the City hospitals of Zurich.

### The interdisciplinary falls prevention program

Since 1998, in-patient falls were systematically registered using the fall incident reporting system. The development and implementation of the fall incident reporting system is described in detail elsewhere [15]. The interdisciplinary falls prevention program (IFP) is designed to provide a safe environment for the hospitalized patients and to reduce the occurrence of falls and consequent injuries It was developed using evidence from an earlier nurse-led fall prevention protocol [29] and literature findings. The IFP protocol consists of three essential elements (Table 1): first, all patients were briefly screened for fall risk as part of the regular nursing assessment upon admission; second, patients considered at risk for falling were examined by a physician; and third, general safety measures and specific interventions to prevent patient falls and subsequent injuries, were routinely implemented.

In 2000, the IFP was introduced in the departments of internal medicine, geriatrics, and surgery. The IFP protocol included 30-minutes of lectures and the provision of the protocol guidelines for nursing staff, physicians, and physiotherapy staff of the participating units. Newly employed personnel were informed "on the job" how to follow the IFP protocol in daily clinical practice. Finally, a falls prevention committee, representing the involved health care professionals was installed to audit the progression of the IFP twice a year.

### Data collection and measurement

The data collection period covered the time before, during and after the implementation of the IFP. Socio-demographic (e.g., age, gender) and clinical characteristics (e.g., length of stay, medical diagnosis) of the studied patients were extracted from the administrative data sets. In-patient falls were reported within 24 hours of occurrence by registered nurses, using the standardized fall incident report form. A fall was defined as "an event in which a patient suddenly and unintentionally came to rest on the floor". Other data collected with the fall incident form were: department, patient demographics, circumstances of the fall, prevalence and severity of injuries, and prevalence of risk factors for falls (i.e. history of falls, impaired mobility, impaired cognition, use of narcotics, and use of psychotropics).

### Statistical analysis

Frequency distributions and summary statistics including proportions, means, and standard deviations were utilized to describe patient characteristics, the prevalence of patient falls and associated characteristics across hospitals departments and years. Fall rates per 1,000 patient days were calculated using falls as the numerator and patient days as the denominator. A general linear model was used to model the rate of falls per 1,000 patient days each 6 months from 1999 to 2003. Demographic and clinical patient characteristics were compared between the clinical departments and between the years under study using Chi-square and analysis of variance as indicated in the tables. All statistics were performed using SPSS for Windows, version 12.0 (SPSS Inc., Chicago, Ill).

## Results

### Patient characteristics

During the study period 36,295 patients were hospitalized, of which 1,323 patients (3.6%) were excluded for further analysis since they were not hospitalized for more than 24 hours in one of the designated clinical departments. In total, 34,972 hospitalized patients were observed (mean age: 67.3, SD ± 19.3 years; female 53.6%, mean length of stay: 11.9 ± 13.2 days, mean nursing care time per day: 3.5 ± 1.4 hours). 11,402 patients aged 80 years and older represented 32.6% of the hospitalized population and accounted for a total of 196,591 patient days (45.6%). The most common of the patient's primary medical diagnoses within the ICD-10 diagnostic categories were as follows: digestive system (19.4%), circulatory system (17.0%), injury/poisoning (13.7%), respiratory system (7.4%), and neoplasm (6.1%).

Half of the patients (49.7%) were hospitalized in the department of medicine, 42.4% in the surgical department, and 7.9% in the geriatrics department, reflecting the size of the departments. Patient characteristics including gender, age, length of hospital stay, and nursing care time per patient differed significantly between the three departments (Table 2).

### Frequencies of in-patient falls

**Figure 1:**
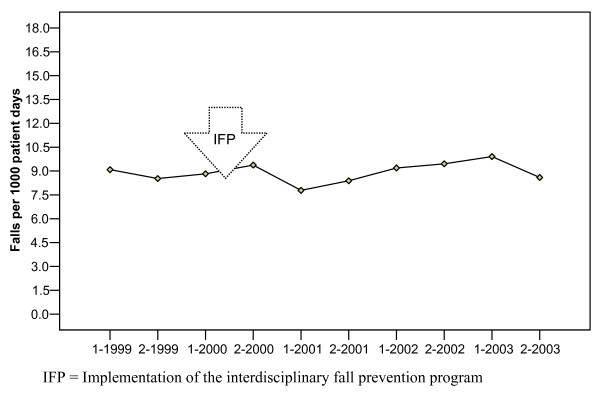
Hospital in-patient fall rates per half year from 1999 to 2003.

Overall, a total of 3,842 falls affected 2,512 (7.2%) of the hospitalized patients. One thousand eight hundred and four (71.8%) patients fell once, 439 (17.5%) fell twice, and 269 (10.7%) fell three times or more. Those patients who fell more than once accounted for 53% (n = 2,038) of all falls. The numbers and percentages of patients who fell per department were 1,538 (8.8%) in medicine, 685 (24.8%) in geriatrics, and 289 (1.9%) in surgery. The overall fall rate was 8.9 falls per 1,000 patient days (geriatrics: 11.7 falls, internal medicine: 11.3 falls, and surgery: 2.9 falls). The fall rates per 1,000 patient days fluctuated slightly from 9.1 falls in the first half of 1999 to 8.6 falls in the second half in 2003. After the implementation of the IFP a slight decrease down to 7.8 falls per 1,000 patient days was observed in the first half of 2001 (Figure 1). However, the observed fluctuations in fall rates over the years under study did not reach statistical significance (p = 0.086). There were no significant differences over time in individual departments (data not presented).

### Severity and type of injuries and evolution over time

From the 3,842 falls, 2,552 (66.4%) remained without injuries, while 1,142 (29.7%) falls resulted in minor injuries (pains, bruises, scratches, haematoma, superficial wounds), and 148 (3.9%) falls resulted in major injuries such as 33 fractures of hands, arms, or ribs, 31 hip fractures, 12 intra cranial bleedings, and 72 other injuries (e.g. luxations, multiple haematoma). The prevalence of minor and major fall related injuries differed significantly in the departments of internal medicine (30.4%, 3.0%), geriatrics (28.0%, 5.0%), and surgery (31.9%, 5.0%) (Chi square, 12.603, df 4, p = 0.013). The prevalence of minor and major fall related injuries differed significantly across the years (Table 3). Fewer minor injuries were observed in 2003 compared to 1999, and more major injuries were observed in 2003 compared to 1999.

### Evolution of patient characteristics from 1999 to 2003

**Figure 2:**
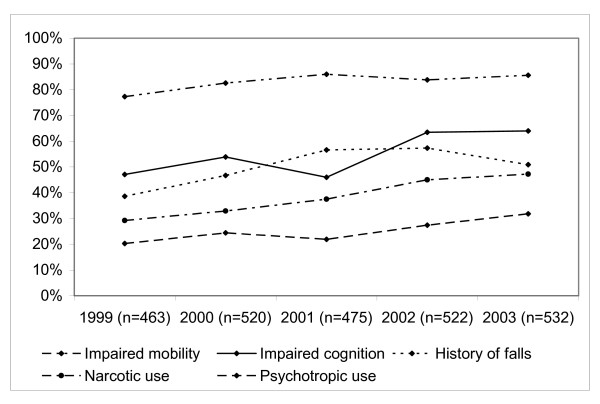
Annual prevalence of risk factors in patients who fell (N = 2,512).

The proportion of female patients from 1999 to 2003 tended to increase from 52.7% to 54.2% (p = 0.235). The mean age of the patients increased from 66.2 ± 19.6 years to 67.8 ± 19.2 years (p < 0.001), and the mean nursing care time per patient increased from 3.4 ± 1.4 to 3.7 ± 1.4 hours per day (p < 0.001) from 1999 to 2003. The mean length of the patient's hospitalization decreased from 12.5 ± 14.7 days in 1999 to 11.7 ± 12.6 days in 2003 (p < 0.001). In those 2,512 patients who fell, the following risk factors were prevalent at the time of their first fall: impaired mobility (83.1%), impaired cognition (55.3%), history of previous falls (50.1%), use of narcotics (38.6%), and use of psychotropics (25.4%). The prevalence of these fall risk factors rose significantly from 1999 to 2003. Impaired mobility increased by 8.3% (p = 0.003), impaired cognition by 16.9% (p < 0.001), use of psychotropics by 11.5% (p < 0.001), and use of narcotics by 18% (p < 0.001), as well as history of falls as a marker for future falls increased by 12.3% (p < 0.001), (Figure 2).

## Discussion

This study examined fall rates, consequent injuries and characteristics of hospitalized patients before and after the implementation of an interdisciplinary falls prevention program. The frequencies of falls, consequent injuries, and clinical patient characteristics varied between the departments of internal medicine, geriatrics and surgery. Following the implementation of the IFP, no reduction of in-patient fall rates and no reduction in consequent injuries were observed within individual departments or in the hospital. During the observation period, the mean length of hospital stay decreased slightly, while the mean nursing care time per patient day increased: both trends may reflect a higher workload for healthcare staff. Additionally, one in three patients was 80 years and older, and in those patients who fell while hospitalized, the prevalence of risk factors for falls increased significantly from 1999 to 2003. These may reflect altered patient characteristics, which lead to proneness to falling.

In this general urban hospital setting, overall fall rates per 1,000 patient days (e.g., 8.9 falls) were higher compared to other studies reporting rates between 2.7 and 4.1 falls per 1,000 patient days [8-10,18,30]. Fall related injuries were seen in 33.6% (3.9% major) of our patients, a proportion that was similar to others reported in the literature [10,18,31]. It appears that irrespective of fall rates, the percentage of patients with consequent injuries remain relatively stable.

Since falls and consequent injuries affect patient safety and may damage a hospital's reputation, various falls prevention programs have been implemented [26,27]. Recently, a 30% and a 28% reduction of falls and subsequent injuries in a sub-acute hospital setting were reported from a randomized controlled trial [32]. These effects were attributed to a targeted multiple intervention program. Another intervention program in elderly patients in a community hospital resulted in a 21% reduction of falls at 6 months postintervention, while no effect was noted for fall related injuries [33]. A falls prevention program in a rehabilitation hospital setting (quasi-experimental study) reported reductions of falls by 15.3%, fewer fallers by 29.7%, and fewer patients with fall related injuries by 51.1% within a 1 year period [34]. Unfortunately, the benefit of the program did not remain significant after correcting for length of stay. In addition, after the implementation of a nurse led falls prevention program in a large general district hospital, fall related injuries were reduced by 25% over a 5-years period, while the number of falls did not change [28]. In another prospective observational study, the intervention effects of ward based quality cycle teams in a rehabilitation hospital resulted in a significant reduction of fall rates per 1,000 patient days comparing 3 years of pre-intervention with 3 years post intervention [35]. In most of these former studies, patients have benefited from falls prevention programs within 6 and 12 months in terms of fewer falls and related injuries [26,32,34], but only two non-experimental studies [28,35] reported positive effects exceeding one year. In the current study neither a sustained reduction of falls nor a decrease in consequent injuries was observed within the 3 years after the implementation of the IFP. This raises questions about whether the interventions of the program was not effective, adherence to the intervention protocol was poor and if the altered patient characteristics may have neutralized intervention effects.

Our study examined the effects of the IFP falls prevention program in daily clinical practice rather than under rigorous research conditions as it was done in other more successful falls prevention studies [32,33]. The IFP consists of the elements reported in intervention studies and falls prevention programs which resulted in reduced fall rates and reduced injury rates. The design of the intervention protocol of the IFP used best available evidence for hospital settings [27,36] and showed positive results in an earlier study [29]. In view of adherence to the protocol, it's assumed from the audits of the falls prevention committee that the physicians and nurses may not consistently practice the IFP. This argument is supported from a study in an acute care metropolitan hospital, with 43% non-adherence with the fall prevention protocol [37]. In another study, compliance with the program deteriorated over time and after 5 years fall rates increased back to the level before the program was implemented [38].

More specifically, in our study data were not available on how often the intervention protocol was followed including screening patients risk for falls and examination of those patients at risk for falls as well as the type of subsequent interventions was applied. This was not the case in another study too [33].

In view of altered patient characteristics it remains unclear if the observed increases in age and decreases of length of stay during the course of the study had an impact on the effectiveness of the program. The relatively high and stable fall rates before and after the IFP may be viewed with regard to a quotation of Bernard Isaacs that "a unit where nobody falls is a unit where nobody moves" [39]. This higher rate may reflect our hospital practice of early remobilization and forced ambulation of the patients in order to reach functional autonomy for hospital discharge as soon as possible. Positive effects of the hospital falls prevention program immediately after implementation may have been caused by an increased initial awareness of nurses rather than by the specific interventions for patients at risk for falling [27,40]. In addition, the IFP was mandated in three different hospital departments each with numerous health care professionals. This approach could be inappropriate for some units since multi-factorial interdisciplinary interventions are often time consuming which may limit their practicability in a busy acute hospital setting.

If clinicians adherence to the intervention protocol was inconsistent, it remains unclear if this can be explained by a lack of commitment on the part of the physicians and the nurses, by insufficient knowledge about which patients were at risk for falling, or whether the high priority given to the acute care treatment of patients contributed to the multifactorial falls risk modification protocol being neglected. The clinicians may not have been adequately prepared and facilitated to integrate the intervention protocol into their daily routine and, therefore, no sustained change of the clinical practice was established. Translating evidence from research into practice remains a challenge. An appropriate approach such as action research [41] should be considered. Action research is basically a self-reflective enquiry undertaken by participants (e.g., clinicians, researchers in hospital settings) in order to improve the rationality and justification of their own practices, their understanding of those practices, and the situations in which the practices are performed [42]. This type of approach may support future attempts to improve interdisciplinary falls prevention practice.

### Limitations

The following limitations of this study have to be considered. First, due to its serial survey design, characteristics of patients and the hospital organization were not controlled. Second, the fall risk profile of those patients who did not fall was not obtained, therefore it was unclear to what extend this population was at risk for falling. Third, adherence to the intervention protocol was not observed or recorded.

The audits may not have been sufficient to ensure sustained adherence to such a complex program because the commitment and clinical expertise of the individual nurses and physicians varied, and may also have been influenced by staffing, patient severity, and communication skills within the interdisciplinary team.

## Conclusion

Following the implementation of an interdisciplinary falls prevention program, neither the frequencies of falls nor consequent injuries decreased substantially. Future studies need to incorporate strategies to maximize and evaluate ongoing adherence to interventions in hospital falls prevention programs.

## Competing interests

The author(s) declare that they have no competing interests.

## Authors' contributions

RS contributed to the conception, design, data collection, analysis, interpretation of data, and drafted the manuscript. HB and SDG contributed to the design, interpretation of data, and critical revision of the manuscript. KM contributed to the analysis, interpretation of data, and manuscript preparation. All authors gave final approval for this version of the manuscript to be published.

## Pre-publication history

The pre-publication history for this paper can be accessed here:



## Figures and Tables

**Table 1:** Components of the interdisciplinary falls prevention program

	**Referring discipline**
**Screening of all patients at admission for risk of falls:**	Primary nurse
- History of falls (i.e. 2 or more falls in the last 6 months)	
- Impaired mobility (e.g., unsteady, weak gait)	
- Impaired cognition (e.g., confused, forgetful)	

**Examination of patients considered at risk for falling:**	Physician
- Note circumstances and consequences of earlier falls	
- Examine patients for acute or chronic medical condition(s)	
- Review medications	
- Assess gait, balance, vision, neurological function, and mental status	

**Interventions for all patients to provide safety in the hospital:**	Primary nurse
- Orient patients to surroundings/"set up" of room	Nursing staff
- Place call bell and personal belongings within reach	
- Keep bed in low position	
- Ensure safe footwear and adequate fit of clothing	
- Provide nightlight at bedside	
- Ensure walking aids (devices) are fitted and used appropriately	
- Lock wheels on wheelchairs, beds, night commodes	

**Interventions in patients considered at risk for falling:**	Physician
- Modification of medication	Primary nurse
- Instruction of patients (family) about risk factors	Nursing staff
- Post fall risk sign in patient's record	Physiotherapy staff
- Assist unsteady patient with ambulating	
- Toilet patient regularly	
- Use half-length side rails instead of full length side rails	
- Exercise program, gait/balance training	
- Provision of hip-protectors	

**Reassessment of those patients who fell:**	Physician
- Evaluation of circumstances and consequences of the fall	Primary nurse
- Reassessment of patient risk factors for falls	
- Continuing or implementation of preventive interventions	

**Table 2:** Patient characteristics

	**Total **(n = 34,972)	**Medicine **(n = 17,386)	**Geriatrics **(n = 2,765)	**Surgery **(n = 14,821)	**P-values**
**Females (%)**	18,745 (53.6)	9,469 (54.5)	2,010 (72.7)	7,278 (49.1)	<0.001†
Age in years*	67.3 ± 19.3	70.4 ± 17.3	83.0 ± 7.8	60.6 ± 20.4	<0.001‡
**Age groups (%)**					
18 – 64 yrs.	36.6	29.2	1.7	51.8	<0.001†
65 – 79 yrs.	30.8	34.2	28.2	27.3	
80 yrs. and more	32.6	36.6	70.1	20.9	
Length of stay(days)*	11.9 ± 13.2	10.8 ± 9.3	36.1 ± 25.4	8.6 ± 8.1	<0.001‡
NCT§(hours)*	3.5 ± 1.4	3.3 ± 1.5	3.7 ± 1.6	3.6 ± 1.3	<0.001‡

*Mean ± SD, ^§^Nursing care time per patient day, ^†^Chi-square, ^‡^ANOVA

**Table 3:** Prevalence of fall related injuries from 1999 to 2003 (N = 3,842 falls)

	**1999**	**2000**	**2001**	**2002**	**2003**	**P-value†**
Number of falls	763	779	689	806	805	
No injuries (%)	64.9	63.4	68.1	67.7	68.1	0.169
Minor injuries (%)	32.6	32.7	26.0	28.9	28.1	0.015
Major injuries (%)	2.5	3.9	6.0	3.3	3.9	0.014

^†^Chi-square
